# Clinical efficacy and imaging outcomes of unilateral biportal endoscopy with unilateral laminotomy for bilateral decompression in the treatment of severe lumbar spinal stenosis

**DOI:** 10.3389/fsurg.2022.1061566

**Published:** 2023-01-06

**Authors:** Yutong Hu, Hao Fu, Dongfang Yang, Weibing Xu

**Affiliations:** ^1^ Graduate School, Dalian Medical University, Dalian, China; ^2^ Department of Spine Surgery, Dalian Municipal Central Hospital, Dalian, China

**Keywords:** unilateral biportal endoscopy (UBE), unilateral laminotomy for bilateral decompression (ULBD), severe lumbar spinal stenosis, clinical outcomes, imaging outcomes

## Abstract

**Objective:**

To investigate the clinical efficacy and imaging outcomes of unilateral biportal endoscopy (UBE) with unilateral laminotomy for bilateral decompression (ULBD) in the treatment of severe lumbar spinal stenosis (SLSS).

**Methods:**

We retrospectively analyzed 50 patients with SLSS treated with UBE-ULBD from October 2018 to March 2021. Visual analog scale (VAS) for back and legs pain, Oswestry disability index (ODI), modified Macnab criteria, complications, hospital stay, preoperative and postoperative dural sac cross-sectional area (DSCA) and Schizas grade, mean angle of facetectomy and osseous lateral recess decompression rate were examined.

**Results:**

The mean follow-up period was 10.7 months. The mean hospital stay was 2.76 ± 1.02 days. At the final follow-up, VAS for back pain and legs pain decreased from 7.22 ± 0.95 to 1.26 ± 0.44 and from 7.88 ± 0.69 to 1.18 ± 0.39, respectively; ODI decreased from 69.88 ± 6.32% to 14.96 ± 2.75%. According to the modified Macnab criteria, the results were excellent in 24 (48%), good in 22 (44%), and fair in 4 (8%). Excellent or good results (a satisfactory outcome) were obtained in 92% of the patients. There were 2 cases of complications of dural sac tear. The postoperative DSCA was significantly enlarged compared with that before surgery, from 44.74 ± 9.85 to 126.86 ± 14.81 mm^2^. According to Schizas grade, the stenosis grade changes from preoperative grade C in 16 cases, grade D in 34 cases, to postoperative grade A in 40 cases, and grade B in 10 cases. The mean angle of facetectomy of the ipsilateral facet joint was 70.87 ± 5.68${}^{\circ }$, contralateral was 65.07 ± 4.98${}^{\circ }$. The decompression rate was 70.81 ± 4.43% (ipsilateral side) and 71.22 ± 3.68% (contralateral).

**Conclusions:**

UBE-ULBD has a good clinical effect in the treatment of SLSS, and has achieved satisfactory results in spinal canal enlargement, undercutting of facet joints, and decompression effect. It is a safe and effective surgical for SLSS.

## Introduction

Lumbar spinal stenosis (LSS) is a degenerative disease of the lumbar spine, common in middle-aged and elderly (1, 2). LSS is usually associated with back pain or sciatica, of which neurogenic intermittent claudication is the typical symptom (3, 4). Anatomically, LSS can be divided into three types, including central spinal stenosis, lateral recess stenosis, and foraminal stenosis (5). Conservative treatment is the preferred treatment for most patients with LSS. In case of failure, surgery should be performed (6).

Presently, severe LSS refers to dural sac cross-sectional area (DSCA) ≤ 75 mm^2^ based on magnetic resonance imaging (MRI) (5), or based on the morphology of the dural sac as Schizas C or D grade (7). And such patients generally have severe osteophyte hyperplasia of facet joints, as well as hyperplasia and even ossification of ligaments， resulting in severe nerve roots or cauda equina compression. Conservative treatment of SLSS has a great risk of failure, and surgical treatment should be actively adopted. Some studies have pointed out that for SLSS, surgical treatment has a better clinical effect than non-surgical treatment (1, 8).

For LSS, traditional laminectomy decompression is a routine surgical procedure (9). This requires extensive resection of the posterior structure and facet joints, which may result in iatrogenic instability and subsequent fusion surgery (9). To reduce the trauma caused by surgery, various minimally invasive techniques have developed rapidly, including endoscopic techniques. However, for SLSS, minimally invasive surgery may not complete decompression due to technical difficulties and other factors (10).

In recent years, unilateral biportal endoscopy (UBE) has generated a wave of frenzied learning. It has an independent working and viewing portal, with the freer operation and higher decompression efficiency, which well overcomes the shortcomings of insufficient decompression in previous endoscopic surgery (11, 12). And the feasibility of UBE with unilateral laminotomy for bilateral decompression (ULBD) in the treatment of LSS has been confirmed to provide satisfactory clinical results (13, 14). In addition, Kim et al. (14) reported the feasibility of UBE technology in the treatment of SLSS, followed up 58 patients, and achieved a satisfaction rate of 93.1%. However, the authors only looked at severe central spinal stenosis in the study and lacked analysis of imaging results. In addition, there were no other reports about this in subsequent studies.

Therefore, this study aims to explore the clinical effect of UBE-ULBD in the treatment of SLSS, and further explore the decompression effect of this technology through imaging results.

## Materials and methods

### Patient population

A total of 50 patients were enrolled from October 2018 to October 2021 in this study. All surgeries were performed by the same senior spine surgeon with extensive experience in endoscopic surgery, who performed >200 surgeries annually.

### Inclusion criteria

- ① Single-segment LSS
- ② Presenting classic neurogenic intermittent claudication with lower extremity symptoms.
- ③ DSCA ≤ 75 mm^2^ or Schizas C or D grade.
- ④ Lumbar spondylolisthesis ≤ I°, and no obvious lumbar instability.
- ⑤ Lumbar scoliosis <20°.

### Exclusion criteria

- ① Multi-segmented LSS
- ② DSCA >75 mm^2^ and Schizas A or B grade.
- ③ Lumbar spondylolisthesis ≥ II° or lumbar instability.
- ④ LSS is caused by fractures, tumors, and infections.
- ⑤ Lumbar curvature ≥20°.
- ⑥ Patients with poor general status or other diseases that cannot tolerate prone surgery under general anesthesia.

### Surgical technique

All patients underwent general anesthesia and were placed in prone position. The abdomen is suspended by placing the arch bridge cushion on the ventral side. The target segment was determined by C-arm fluoroscopy.

The incision design was as follows (for right-hander): (1) the incision on the left: 5 mm aside the posterior midline, the working portal (8–10 mm) was located at the upper edge of the lower lamina, and the view portal (4–6 mm) was located about 25–30 mm above the working portal (according to the soft tissue thickness). (2) The incision on the right: 5 mm aside the posterior midline and moved slightly caudally compared to the left.

Firstly, the stripper can be used to detach the multifidus from the SP base and lower edge of the upper lamina. Then the trocha was placed directing at the conjunction between the SP base and the upper lamina. A cruciate incision is made into the deep fascia of the working channel to maintain a fluent outflow. After expanded by the serial dilators, the endoscopy and instrument were inserted into the two portals respectively. Radiofrequency was used to clean the surrounding soft tissue, an “initial camp” was created as the working space.

Then resect anticlockwise from the lower edge of the upper lamina, the medial portion of the inferior articular process (IAP), the superior articular process (SAP) and the upper edge of the lower lamina. During this procedure, resect the fluffy superficial layer of the ligamentum flavum (LF) first to obtain a better view. Then expose the attachment of the LF and remove the medial part of the SAP under the lateral margin of the dura was seen. Then perform the lateral recess decompression with curette and rongeur. Decompression was supposed to be enough until the medial border of the pedicle was observed.

**Figure 1:**
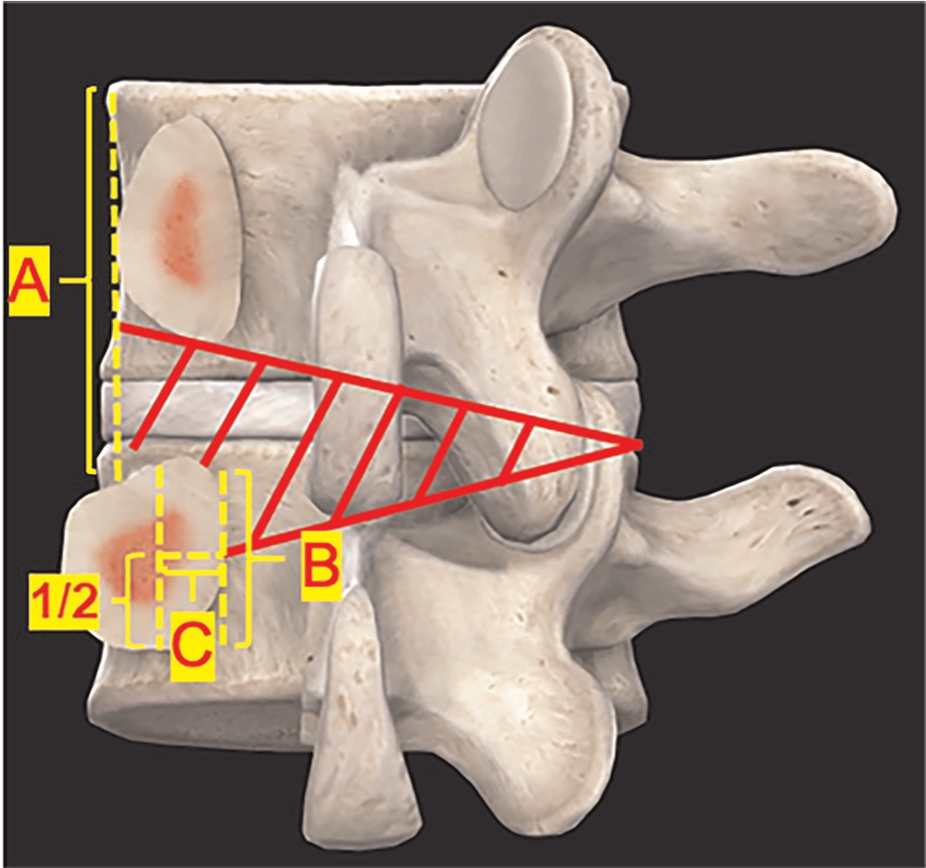
Secompression range of unilateral biportal endoscopy. (**A**) Outer border of the pedicle; (**B**) Inner edge of pedicle; (**C**) Midline border of the pedicle. The contralateral decompression extent (red area). Lateral recess decompression should reach half of the medial border of the pedicle (**C**), and the foraminal decompression should reach the outer border of the pedicle (**A**).

After ipsilateral decompression is satisfactory, resect part of the ventral bone structure (about 5 mm) of both upper and lower SP base to entry the contralateral canal. Herein, the central fissure of the LF (also called “V collar”) can be regarded as a landmark of midline of the canal. Move over the deep layer of the LF, resect part of the ventral surface of the lamina (about 3–4 mm) until the contralateral IAP was seen. Remove part of the IAP (about 5–6 mm) to reveal the SAP. The SAP removal was enough until the medial border of the pedicle was recognized. If needed, then the contralateral lateral recess decompression and foraminal decompression of both segments can be performed simultaneously. The ideal contralateral decompression extent was showed in Figure 1.

**Figure 2:**
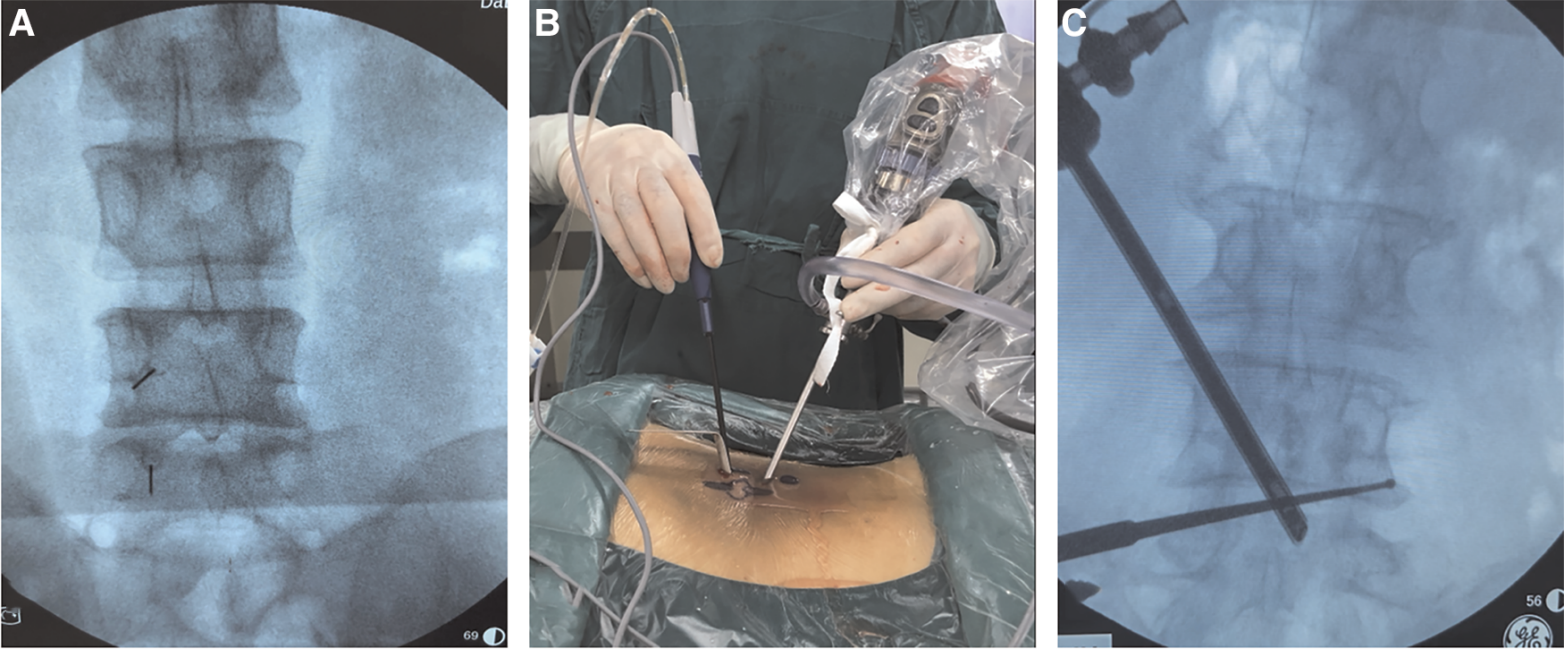
Operation and localization of unilateral biportal endoscopy. (**A**) Pictures of surgical procedures, the left hand of the surgeon is the endoscope, and the right hand is the radiofrequency probe; (**B**) 5 ml syringe needles were used for preoperative incision localization; (**C**) A nerve root probe was used to explore the foraminal region to determine the extent of decompression.

**Figure 3:**
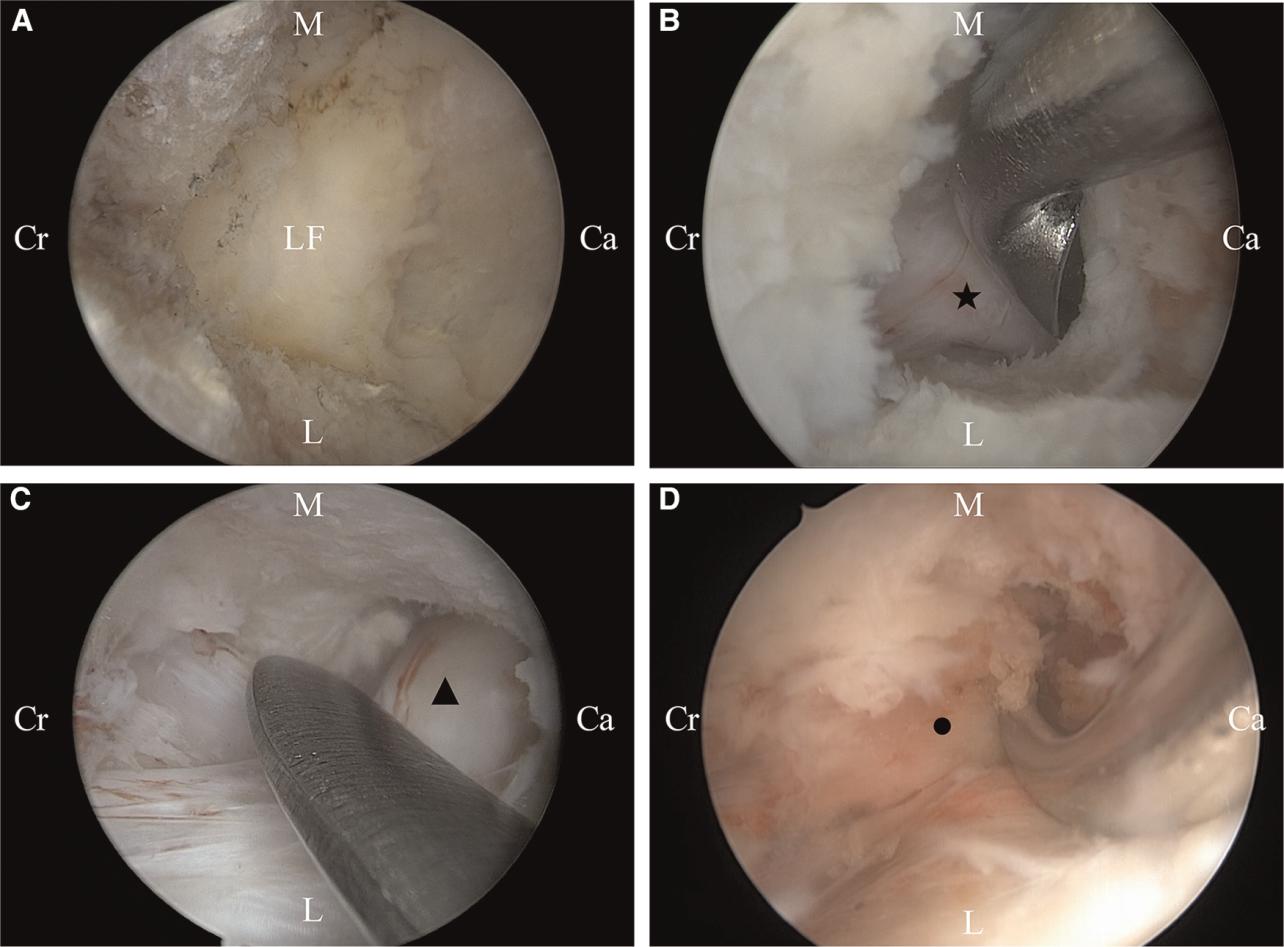
Intraoperative conditions of unilateral biportal endoscopy for decompression. (**A**) Initial camp; (**B**) Decompression of the ipsilateral traversing nerve root; (**C**) Decompression of the contralateral traversing nerve root; (**D**) Decompression of the contralateral foraminal. Ca, caudal sides; Cr, cranial sides; L, lateral; M, medial. LF, ipsilateral traversing nerve root; ▴, contralateral traversing nerve root; ●, contralateral exiting nerve root.

Then, the instrument was moved cephalically and the nerve roots in the foraminal region were probed for compression. The tip of the SAP and the LF in the foraminal region were removed. The foraminal region was decompressed to the outer edge of the pedicle. After satisfactory decompression, the disc on the ventral side of the dural sac was probed for disc herniation. If disc herniation was detected, the herniated nucleus pulposus was removed. The drainage tube was placed under endoscopic guidance (Figures 2, 3).

### Evaluation

The hospital stays and complications of all patients were recorded. All imaging examinations were completed in all patients before surgery. Computed tomography (CT) examination was performed within 3 days after surgery and MRI examination was performed within 1 month after surgery.

VAS for back and leg pain were used to assess the severity of back and both legs pain before surgery, 1 day and 1 month after surgery, and during the last follow-up. The ODI was used to assess low limb dysfunction before surgery, 1 month after surgery, and during the last follow-up. The modified Macnab criteria were used to examine patient satisfaction at the last follow-up.

**Figure 4:**
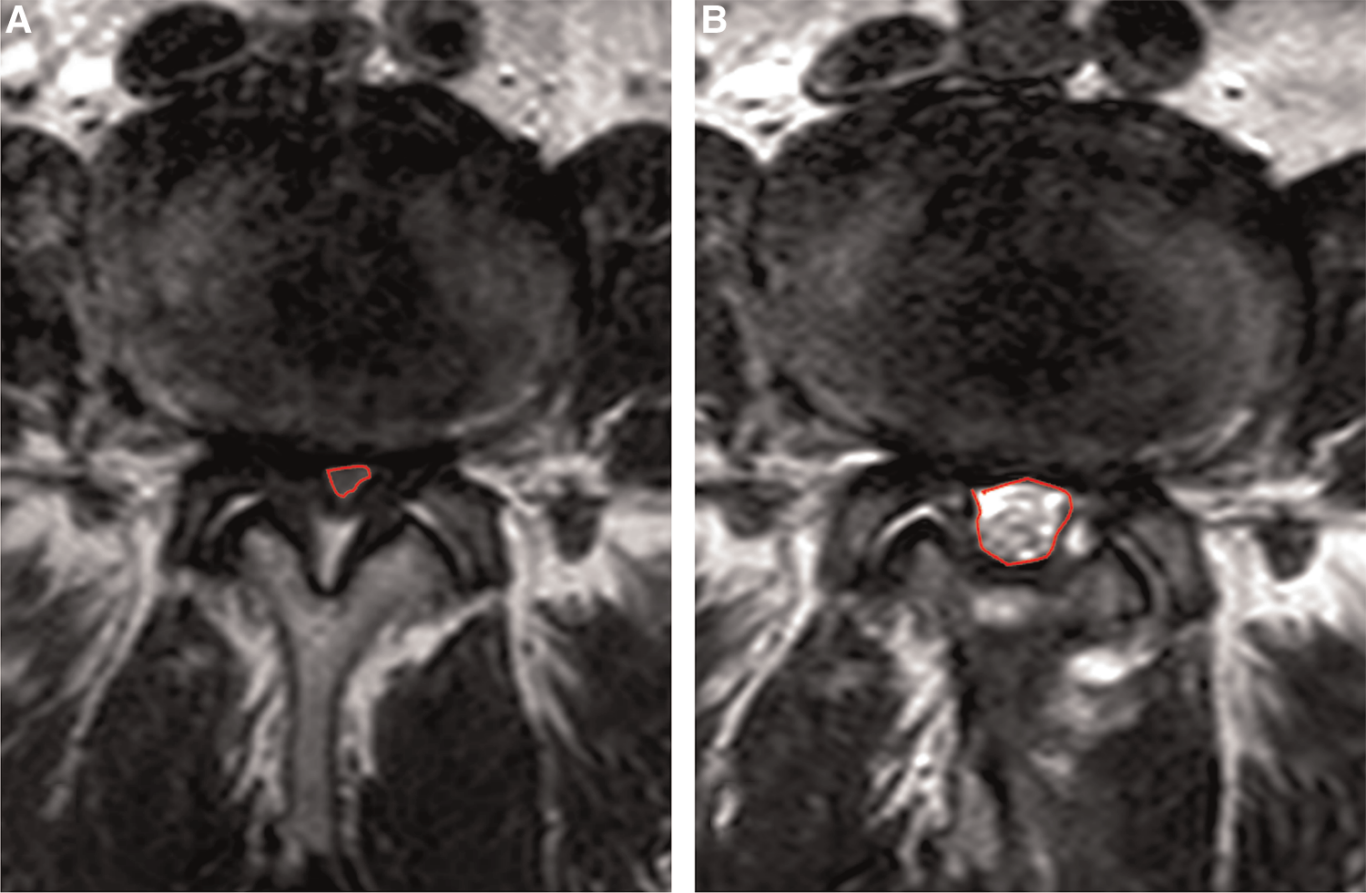
Dural sac cross-sectional area calculation under MRI. (**A**) Preoperative calculation; (**B**) Postoperative calculation.

In terms of imaging outcomes, three consecutive images were taken by two independent imaging doctors at the level of the intervertebral disc with an interval of 3 mm at axial T2-weighted MRI. DSCA was calculated by describing the boundary of the dural sac (Figure 4), and the degree of spinal stenosis was evaluated according to Schizas grade by observing the morphology of the dural sac.

**Figure 5:**
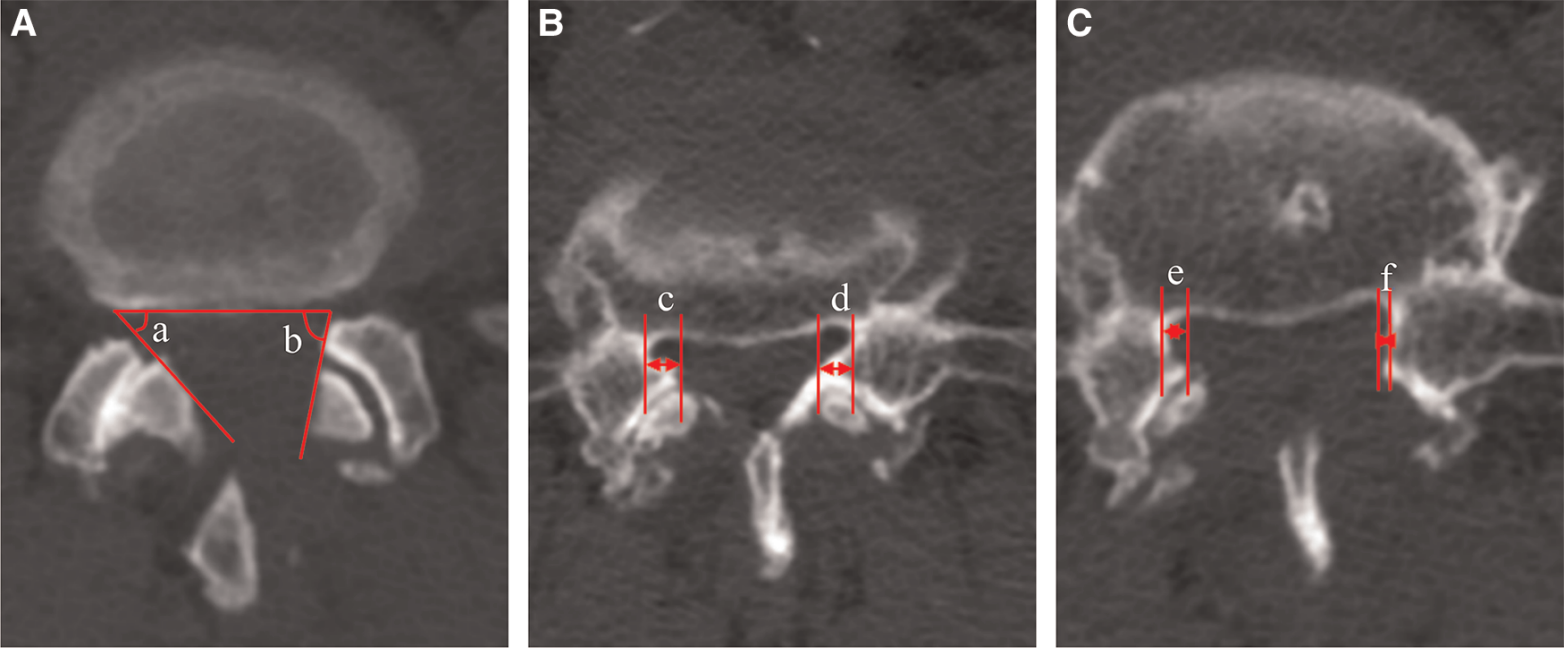
Measurement of resection angle of facet joint and decompression rate under CT. (**A**) Resection angle of facet joint, (**a**) contralateral side, (**b**) ipsilateral side; (**B**) The preoperative distance from the inner edge of the superior articular process to the inner edge of the pedicle, (**c**) contralateral side, (**d**) ipsilateral side; (**C**) The postoperative distance from the inner edge of the superior articular process to the inner edge of the pedicle, (**e**) contralateral side, (**f**) ipsilateral side.

The mean angle of facetectomy was calculated by measuring the angle of the inner facet of the articular process to the axial horizontal line under postoperative CT to evaluate the retention of the facet joint. The osseous lateral recess decompression rate was calculated by the formula (1- the postoperative distance from the inner edge of the SAP to the inner edge of the pedicle under CT/the preoperative distance)∗ 100%, to assess the imaging decompression effect. To minimize the error caused by inconsistent plane selection. Three cross sections were taken consecutively at the target level and measured respectively. The results were added and averaged. (Figure 5).

All radiology-related measurements and calculations were performed by two independent radiologists. All data were recorded independently by three clinicians, and the average was considered for statistical analysis.

### Statistical analysis

SPSS26.0 statistical software was used for statistical analysis. The data are presented as mean and standard deviation. Paired sample t-test was applied for intragroup comparison. *P *< 0.05 indicated a statistically significant difference.

## Results

In this study, a total of 50 patients were enrolled, including 20 males and 30 females, with a mean age of 68.52 ± 7.03 years. The lesion segments were L3/4 in 16 patients, L4/5 in 31, and L5/S1 in 3 (Table 1).

**Table 1:** General condition and surgical indicators of subjects.

	*N* = 50
Sex (males/females)	20/30
Age (years)	68.52 ± 7.03
**Lesion segment [*n* (%)]**	
L3–4	16
L4–5	31
L5–S1	3
Operation time (min)	60.16 ± 7.50
Blood loss (ml)	34.24 ± 15.13
**Surgical complications** (***n***)	
Dural tear	2
Nerve root injury	0
Infection	0
Transfusion [*n* (%)]	0 (0)
Time in bed (day)	1.62 ± 0.75
Hospital stays (day)	2.76 ± 1.02

### Surgical indicators and clinical outcomes

The mean follow-up period was 10.7 months (minimum duration of 6 months). The mean hospital stay was 2.76 ± 1.02 days. 2 patients developed symptoms of dural sac tear, which were gradually relieved after conservative treatment and recovered completely within 5 days. (Table 1).

The VAS for back pain and legs pain was significantly improved from day 1 after surgery and decreased from 7.22 ± 0.95 to 1.26 ± 0.44 and from 7.88 ± 0.69 to 1.18 ± 0.39, at the final follow-up, respectively. ODI improved from 69.88 ± 6.32% to 14.96 ± 2.75% at the final follow-up. According to the modified Macnab criteria, the results were excellent in 24 patients (48%), good in 22 (44%), and fair in 4 (8%). Excellent or good results (a satisfactory outcome) were obtained in 92% of the patients. (Table 2).

**Table 2:** Clinical outcomes in diffident times.

Time	VAS score for back pain	VAS score for legs pain	ODI (%)
Preoperative	7.22 ± 0.95	7.88 ± 0.69	69.88 ± 6.32
Postoperative 1 day	3.00 ± 0.64	3.28 ± 0.78	
Postoperative 1 month	2.56 ± 0.58	2.18 ± 0.56	30.96 ± 3.57
Final follow-up	1.26 ± 0.44	1.18 ± 0.39	14.96 ± 2.75
*F* value	489.913	1153.332	1703.927
*P* value	0.000	0.000	0.000

VAS, visual analog scale; ODI, oswestry disability index. *P *< 0.05 considered as significant.

### Imaging outcomes

The postoperative DSCA was significantly enlarged compared with that before surgery, from 44.74 ± 9.85 to 126.86 ± 14.81 mm^2^. According to Schizas grade, the stenosis grade was improved from preoperative grade C in 16 cases and grade D in 34 cases to postoperative grade A in 40 cases and grade B in 10 cases. The mean angle of facetectomy of ipsilateral facet joint was 70.87 ± 5.68${}^{\circ }$, contralateral was 65.07 ± 4.98${}^{\circ }$, both less than 90${}^{\circ }$. The decompression rate of the osseous lateral recess on the ipsilateral side was 70.81 ± 4.43%, and the contralateral was 71.22 ± 3.68%. (Table 3).

**Table 3:** Imaging outcomes.

	UBE
**DSCA (mm^2^)**	
Preoperative	44.74 ± 9.85
Postoperative	126.86 ± 14.81
**Schizas grades (*n*)**	
Preoperative (A/B/C/D)	0/0/16/34
Postoperative (A/B/C/D)	40/10/0/0
**Mean resection angle of facet joint (** ${}^{\circ }$ **)**	
Ipsilateral	70.87 ± 5.68
Contralateral	65.07 ± 4.98
**Osseous lateral recess decompression rate (%)**	
Ipsilateral	70.81 ± 4.43
Contralateral	71.22 ± 3.68

DSCA, dural sac cross-sectional area.

## Discussion

SLSS is usually associated with central spinal stenosis, lateral recess stenosis, and foraminal stenosis, with multiple nerve root compression (15). Presently, the main purpose of surgical treatment for SLSS is to fully decompress and relieve nerve compression.

In this condition, traditional open laminectomy for whole spinal canal decompression is a preferer option. However, the large trauma and additional fusion and fixation lead to slow recovery, more complications, and further adjacent segment degeneration (9, 10). Some studies have shown that in patients (>65 years old) with SLSS, the probability of life-threatening complications (including mortality) during surgery increases with trauma (14). The ULBD technique has been proven effective and minimally invasive (retains 80% lumbar stiffness) but has not been widely performed by its technical difficulty under microscopy or full endoscopy, especially in SLSS cases (16–18). ULBD under uniportal endoscopic (UE) for LSS decompression also has been reported (13, 19, 20). The common advantages, like less trauma, less bleeding, lower complication rate and faster recovery has been proven (21). However, the hard coaxial confine the viewing and working scope, unfree manipulation results in risk of insufficient decompression. In addition, specialized surgical instruments and high learning curve limit its wide application further (22–25). However, studies showed that decompression of SLSS by ULBD under microscopy and full endoscopy may have risks such as insufficient decompression and increased complication rate (10, 15, 26). While because of the independent view-working channel, unlimited operation angle, more instruments option, and better visualization, UBE was thought to be optimal for UBLD, even for SLSS cases (14, 27). Chio et al. (9) pointed out that UBE has achieved good clinical efficacy in the short-term follow-up for the treatment of LSS and this technology has the advantages of less trauma, less bleeding, quick recovery, and a short learning curve.

Multifidus is the most easily injured paravertebral muscle in posterior spinal surgery and is only innervated by the medial branch of the posterior ramus of the spinal nerve (28). The dissection and contraction of the paravertebral muscle can lead to muscle denervation and atrophy, increasing the risk of back surgery failure (29). UBE creates working space through natural lacunae such as the multifidus triangle, which can effectively reduce the damage to the paravertebral structure (such as multifidus). The results of this study show that VAS scores for back and legs pain were significantly improved immediately after surgery and continued to improve during follow-up. At the final follow-up ODI was improved from 69.88 ± 6.32% to 23.28 ± 3.87% and 92% of patients have excellent or good outcomes according to the modified Macnab criteria. The lower postoperative back pain may be due to less trauma to the multifidus muscle, and the relief of postoperative leg pain also reflects the better effect of nerve root decompression.

Facet joints, as part of the “three-joint complex” of the spine, are extremely important in maintaining spine stability. Thus, extensive resection of facet joints may lead to postoperative segmental instability (11, 30). Studies have demonstrated that preservation of facet joint integrity can reduce postoperative instability and avoid additional fusion (18). In this study, the ipsilateral and contralateral mean facetectomy angles were both less than 90${}^{\circ }$ which even smaller than the previous study which reported a mean facetectomy angle <90${}^{\circ }$ indicating better preservation of facet joints (25). We thought that may be because of the special view of 30-degree endoscopy which allows an easier undercutting than 0-degree endoscopy (14). Pao et al. pointed out that in SLSS cases, due to the structural malformations and narrow space, full decompression may need more damage to the ipsilateral facet joint. To deal with this problem, performing contralateral decompression first or resecting more bone of the SP base shall be suitable (11).

DSCA and Schizas grade is commonly used in the clinical evaluation of the degree of spinal stenosis. In this study, the postoperative DSCA was significantly enlarged and the degree of spinal stenosis was recovered to grade A or grade B postoperatively. Considering the individual differences of spinal canal size in different patients, we developed the decompression rate of the osseous lateral recess and other detection parameters according to the pathological factors of stenosis to evaluate the decompression effect of lateral recess. However, this parameter has not been mentioned in the international community, and the rationality of this parameter needs to be further explored and verified by large sample data.

In terms of decompression strategy, considering the complexity of severe lumbar spinal stenosis, there may be central spinal stenosis, lateral recess stenosis, and foraminal stenosis at the same time, so they are generally separated during decompression. For central spinal stenosis, partial laminectomy and resection of the ligamentum flavum are required to achieve enlargement of the spinal canal, and management of the ventral disc herniation of the dural sac is required (5). In the management of lateral recess stenosis, adequate excision of the inner edge of the SAP and IAP is necessary, but excessive excision should be avoided to affect the stability of the spine (11, 30). For foraminal stenosis, the tip of the SAP should be removed and the ligaments in the foraminal area should be fully released. The SAP should be removed in pieces. The excised area should be <50% of the SAP. If it is >50%, the translational and rotational stabilities of the movable segment may be affected (31). In addition, avoiding nerve root damage and protecting the radicular artery during decompression is essential.

For decompression extent, Wang et al. (32) clarified the definition of lumbar lateral recess and proposed the West China classification, dividing the lumbar lateral recess into 6 zones. The study also pointed out that the degeneration occurs mostly in zones 1, and 2 (about 81.5%). Wherein, zone 2A is the most frequent site of nerve root compression. As for foraminal stenosis, Murata et al. pointed out that stenosis was mainly concentrated at the outer edge of the pedicle (outside the pedicle's center), reaching 94% (33). Therefore, in UBE-ULBD for SLSS, the ideal decompression extent shall be: (1) transversely, the inner edge of the bilateral pedicle was used as the boundary. (2) longitudinally, the proximal decompression reached the ventral surface of the cephalad lamina (the cranial attachment of the LF) and the distal decompression reached the midline of the pedicle to achieve sufficient decompression of zone 2 (Figure 5).

In this study, 2 patients developed dural sac tears and no patients developed an infection. Continuous irrigation with saline helps control bleeding and reduce postoperative infection (34). To avoid intracranial hypertension caused by high-pressure saline, controlling the saline pressure at 20–30 mmHg is recommended. The total dural tear rate reported in UBE was 1.9%–5.8% and occurred mostly on the dorsal side, caused by water pressure, instrument friction, limited field of vision, difficulty in anatomical identification, and the learning curve (35). It has been reported the dural tear rate significantly decreases after 50 surgeries (36). In addition, in SLSS, calcification or ossification of the LF sometimes may adhere to the dural sac, as well as the central folding of the dura sac hidden in epidural fat tissue. According to the preoperative MRI and intraoperative observation, we found that epidural fat was significantly reduced or even disappeared. Therefore, it is supposed to be more careful to identify the adhesion and separate the dural sac and LF.

Nevertheless, the present study has several limitations. First, this study lacked a control group, which led to the failure to fully clarify the advantages and disadvantages of UBE in the treatment of SLSS. Second, this was a retrospective study with small sample size and short follow-up time, which failed to evaluate the long-term efficacy of patients.

## Conclusions

UBE-ULBD is a safe and feasible surgical method in the treatment of SLSS. Due to its unique technical advantages, UBE-ULBD can achieve satisfactory whole spinal canal decompression. Meanwhile, it preserves more stabilization structures of the spine to the greatest extent and avoids further fusion and internal fixation. It has the advantages of less trauma, less bleeding, faster recovery, lower complication rate, and lower operation cost.

## Data Availability

The original contributions presented in the study are included in the article/Supplementary Material, further inquiries can be directed to the corresponding author/s.
